# Exploring the impact of waiting time on physical function and psychosocial measures in individuals with lower limb osteoarthritis awaiting orthopaedic consultation

**DOI:** 10.1177/13591053251375313

**Published:** 2025-11-02

**Authors:** Alev Asilioglu, Sara Vogrin, Mary N. Woessner, Jack Behne, Alexander Tacey, Michaela Pascoe, Phong Tran, Carlie Bauer, Loandy Jourdan, Mary De Gori, Catherine M. Said, Michael J. McKenna, Vasso Apostolopoulos, Alexandra G. Parker, Itamar Levinger, Rhiannon K. Patten

**Affiliations:** 1 Victoria University, Melbourne, VIC, Australia; 2 The University of Melbourne, Parkville, VIC, Australia; 3 Victoria University, University of Melbourne and Western Health, Melbourne, VIC, Australia; 4 Western Health, Footscray, VIC, Australia; 5 Southwest University, Chongqing, China; 6 Zhuhai College of Science and Technology, China

**Keywords:** osteoarthritis, depression, social connectedness, loneliness, physical function

## Abstract

Osteoarthritis (OA) is a muscular condition that often requires treatment by orthopaedic specialists leading to length waiting periods. The study aim was to examine associations between waiting time, physical function and psychosocial variables in individuals with lower limb OA awaiting orthopaedic consultation. Data were collected from 80 individuals. Physical function assessments included 6-minute walk test, timed up-and-go, chair stand test, and handgrip strength. The UCLA Loneliness Scale, Social Connectedness Scale, and Patient Health Questionnaire-9 questionnaires were used. Linear or ordinal regressions were used to determine associations. Greater wait time was associated with higher depression symptoms (β = 1.03, 95% CI: 1.01, 1.06, *p* = 0.015), but not social connectedness, loneliness, or physical function. Higher depression scores were related to increased loneliness (*r* = 0.582, *p* = <0.001), and lower social connectedness (*r* = −0.470, *p* = <0.001). Increased wait times are associated with elevated depression scores in individuals with lower limb OA. Shortening wait time may reduce the likelihood of developing depression.

## Associations between waiting time, physical function and psychosocial measures in individuals with lower limb osteoarthritis waiting for orthopaedic consultation

Osteoarthritis (OA) is associated with chronic pain and physical limitations and can negatively impact mental health, quality of life, and everyday living activities, including socialising. Lower limb OA often requires treatment by an orthopaedic specialist leading to lengthy periods on waitlists in the public health system. Although, there is a known link between physical function, depression, loneliness and social connectedness, it is unclear whether waiting time impacts these factors.

The aim of the study was to determine whether associations are present between waiting time and physical function and social connectedness, loneliness and depression symptoms in individuals with lower limb OA on the waitlist for orthopaedic services.

Data were collected from 80 individuals with OA (knee = 78%, hip = 19%, and both = 3%). Physical function assessments included 6-minute walk test (6MWT), timed up-and-go (TUG), chair stand test (CST), and handgrip strength. The UCLA Loneliness Scale, Social Connectedness Scale (SCS), and Patient Health Questionnaire-9 (PHQ-9) questionnaires were used. Linear or ordinal regressions were used to determine associations between wait time and physical function and psychosocial questionnaire outcomes. Age, sex, OA location (hip, knee, both or bilateral), and education level were included as confounders.

Greater wait time was associated with greater depression symptoms (β = 1.03, 95% CI: 1.01, 1.06, *p* = 0.015), but not social connectedness (β = −0.09, 95% CI: −0.25, 0.08, *p* = 0.299), loneliness (β = 1.01, 95% CI: 0.97, 1.06, *p* = 0.299), or physical function; 6MW (β = −2.84, 95% CI: −6.47, 0.80, *p* = 0.122), TUG (β = 1.01. CI: 0.99, 1.02, *p* = 0.227), CST (β = −0.03, 95% CI: −0.14, 0.07, *p* = 0.517), handgrip strength (β = −0.28, 95% CI: −0.68, 0.11, *p* = 0.151). Higher depression scores were related to increased loneliness (*r* = 0.582, *p* = <0.001), and lower social connectedness (*r* = −0.470, *p* = <0.001).

Increased wait times are associated with elevated depression scores for individuals with lower limb OA. Shortening wait time may reduce the likelihood of developing depression, which in turn may be related to improved social interactions.

## Introduction

Osteoarthritis (OA) is a common and multifaceted musculoskeletal condition that involves the alteration of joint tissues, both anatomically and physiologically (Allen et al., 2022). The condition can cause persistent pain, physical disability, and can impact mental health, quality of life, and the ability to perform everyday living activities, including participating within a social network (Alabajos-Cea et al., 2021; Australian Institute of Health and Welfare, 2019; Fonseca-Rodrigues et al., 2022; Jackson et al., 2020; Kraus et al., 2019; Stamm et al., 2016). Across Australia, approximately 9.3% of the population have OA, with the prevalence increasing amongst the female sex (Australian Institute of Health and Welfare, 2019). OA has no cure, rather the condition requires management and treatment from specialists either through conservative or surgical interventions (Davidson et al., 2024; Lim and Al-Dadah, 2022; Naylor et al., 2022). Conservative management may include several approaches including exercise, OA education, healthy eating and weight management, and pain management skills (Lim and Al-Dadah, 2022). While conservative management of lower limb OA is recommended, surgery remains a popular option, particularly for late-stage OA (The Royal Australian College of General Practitioners, 2018). This can lead to lengthy periods on waitlists in the public health system, both for the initial surgical consult and the surgery itself. The average wait time for total joint replacement surgery in Australia is approximately 14 to 15 months (Australian Institute of Health and Welfare, 2023). During this waiting period, patients receive or engage with minimal support (Davidson et al., 2024).

Being on a waitlist leaves patients uncertain of when they will receive orthopaedic care and thus forces people to navigate an ambiguous interim phase during which they engage with life differently than they normally would (Parsons et al., 2009; Sjöling et al., 2005). Waitlists can impact patients both physically and psychologically (Ackerman et al., 2011; Addai et al., 2024; Ho et al., 2021). From a physical health perspective, pain has been reported to either remain stable or worsen while on the waitlist (Desmeules et al., 2010; McHugh et al., 2008; Patten et al., 2022). Similarly, physical function may also deteriorate while on a waitlist (Desmeules et al., 2009, 2010; Kapstad et al., 2007; McHugh et al., 2008; Ostendorf et al., 2004). From a psychological perspective, waitlists are associated with a decrease in quality of life and an increase in psychological distress, with a third of patients experiencing depression (Ackerman et al., 2011, 2005; Patten et al., 2023). Physical limitations and mental health concerns are further associated with poor social relationships (Perruccio et al., 2021; Santini et al., 2020).

Social connectedness refers to the presence and level of integration an individual has with their social network and can encompass a sense of belongingness and quality relationships (Asante and Karikari, 2022). Loneliness refers to dissatisfaction over the absence of social connectedness or quality social relationships (Smith et al., 2019). Loneliness has been found to impact health both physiologically and psychologically, with studies suggesting an increased risk of frailty, and developing cardiovascular diseases, and depression (Bevilacqua et al., 2021; Kojima et al., 2022; Luo et al., 2012; Shankar et al., 2011). The key risk factors that contribute to the often co-occurring outcomes of loneliness, depression, physical limitations, and lower levels of physical activity are being female, being younger in age, having lower levels of education, having greater levels of pain, having multiple joints impacted by OA, or having comorbid health conditions (Buchman et al., 2009; Hawkley et al., 2009; Philip et al., 2020; Shankar et al., 2011; Siviero et al., 2020; Vancampfort et al., 2019; Wood et al., 2016; Zheng et al., 2021). As physical activity and exercise are considered first-line treatment options for the conservative treatment for lower limb OA, addressing factors such as loneliness and social connectedness, may help enhance adherence to exercise programs (Bannon et al., 2021).

Although the literature acknowledges a connection between physical function, depression, loneliness, and social connectedness, the impact of being on a waitlist on these factors remains unclear. The aim of the present study was to determine whether a longer waiting time is associated with a decrease in physical function and social connectedness and an increase in loneliness, and depression in people with lower limb OA on the waitlist for orthopaedic services. The secondary aim of the study was to investigate the inter-relationships between loneliness, social connectedness and depression, with the additional focus of sex as a variable.

## Methods

The current study was conducted as part of a larger research project called the Waitlist Project that aimed to investigate the health and wellbeing of patients on the orthopaedic waitlist to ascertain an understanding of their needs and develop appropriate interventions. Participants were recruited from the orthopaedic waitlist at Western Health, a public hospital located in the western suburbs of Melbourne, Australia. This is an indefinite waitlist where the patient does not have an indication of when they will receive an appointment. Recruitment commenced in August 2021 and continued until April 2022. The inclusion criteria for the wider Waitlist Project were individuals with OA in either the hip and/or knee who were on the orthopaedic waitlist between the 1st of January 2018 and the 1st of June 2022 and were awaiting initial consultation with a specialist, who were over the age of 18 years, who were able to provide informed consent and who consented to being contacted for research purposes. Individuals were considered to have OA based on their referrals or suspected to have OA based on their responses to the three questions listed below, with affirmative responses to all three questions being an indicator of OA:

1. Are you over the age of 45?
2. Do you ever experience activity-related joint pain?
3. Do you either have no morning joint-related stiffness OR have morning stiffness that lasts no longer than 30 minutes?

The exclusion criteria for the study were individuals under the age of 18, those that did not have OA, those unable to provide informed consent, other clinical reasons (such as being a high falls risk), those that had been previously screened by team members and were not fit for testing (which refers to patients that were unable to complete at least one of the physical assessments listed below), those that declined to be contacted for future research purposes, and those that had a specialist appointment booked when initially contacted. The screening assessment undertaken for this study matched the standard assessment utilised by the Western Health physiotherapy clinic. The Strength, Assistance with walking, Rise from a chair, Climb stairs, and Falls questionnaire (SARC-F) was also used as an additional safety screening, with participants not proceeding to the physical assessments if they had had more than four falls in the past year or scored two in the falls subsection (Ishida et al., 2021).

### Procedures

**Figure 1.**
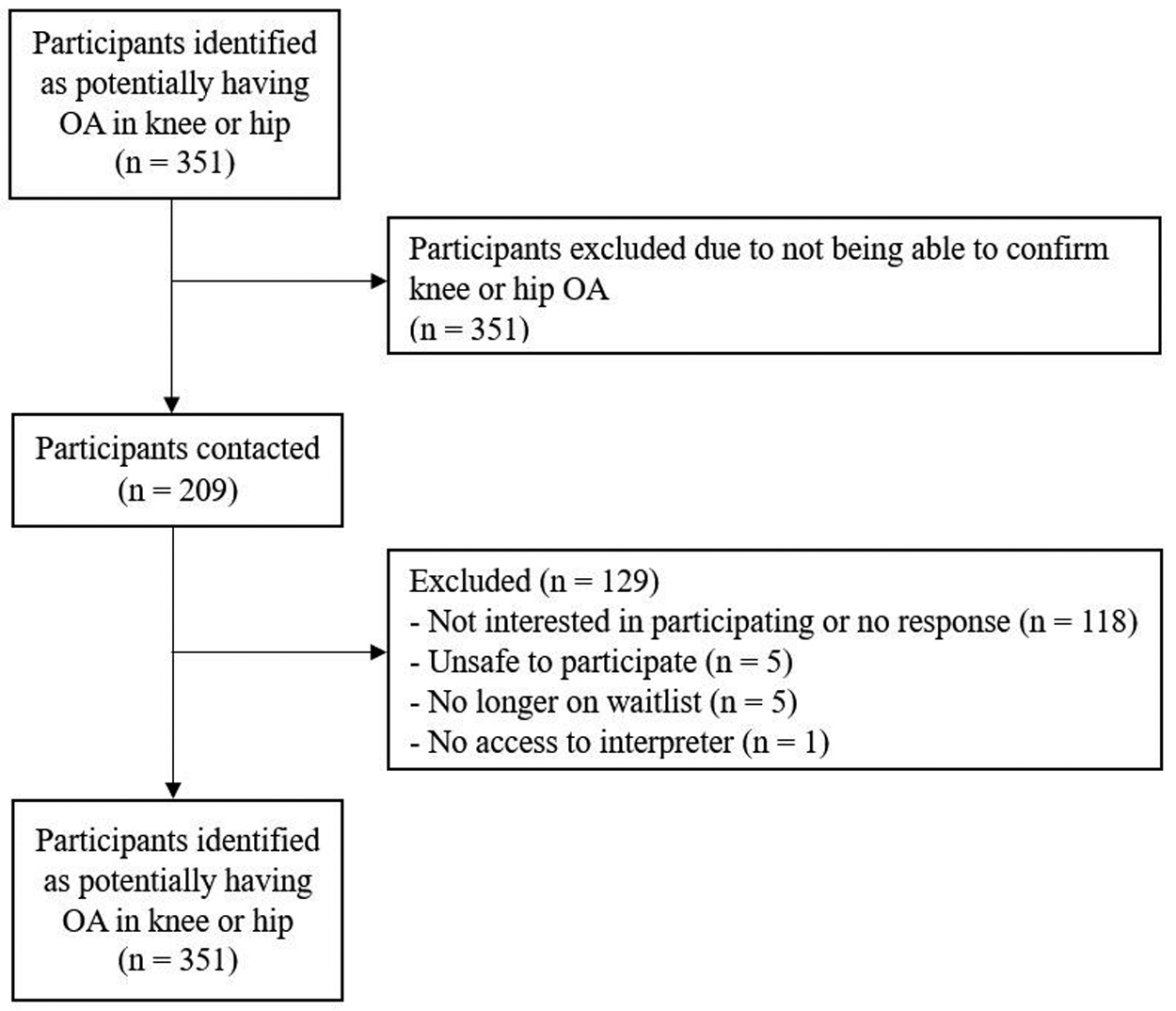
Participant flow diagram.

Melbourne Health Human Research Ethics Committee (2021.055) approved the study and Western Health granted site specific approval. Victoria University Human Ethics Committee provided mirrored ethical approval. Individuals who met the criteria were contacted via a telephone call. Patients who did not respond after three attempts were marked as non-responders and excluded from the study. Those interested in the study were sent a survey package via email, mail, or were completed over the phone. The email contained a link to a secure website (REDCap) where participants could complete the surveys that collected demographic information and the psychosocial questionnaire data. Hard copies were mailed to participants and contained an addressed and prepaid envelope to return the surveys. Surveys that were completed over the phone involved the questions and answers being read aloud with the response being recorded on the participant’s behalf. Prior to data collection, informed consent was obtained (Figure 1).

### Physical assessments

Social distancing measures implemented due to the COVID-19 outbreak required several of the physical assessments to be performed online. The procedures for the physical assessments varied between the face-to-face and the telehealth format. The assessments were carried out by a Western Health physiotherapist. Prior to data collection, participants underwent a general health screening by a Western Health physiotherapist to confirm the presence of lower limb OA and ensure all participants were of a low falls risk and were considered safe to proceed with the study. All the physical assessment data for each participant was gathered during a single assessment.

### Face-to-face physical assessments

Body weight and height were collected. Body mass index (BMI) was calculated using the standardised equation.

The 6-minute walk test (6MWT) required participants to walk for 6 minutes between two markers set 15 m apart (ATS Committee on Proficiency Standards for Clinical Pulmonary Function Laboratories, 2002). The participants were instructed to walk safely as far as possible within the 6-minute timeframe.

The timed up-and-go (TUG) assessment required the participant to stand up from a standard armchair (~46 cm), walk 3-m, turn around and return to a seated position in the chair (Christopher et al., 2021). The best of three attempts was recorded.

The chair stand test (CST) required the participant to rise from a standard armchair to an upright standing position with the arms crossed and held against their chests before returning to a seated position as many times as possible within 30 s (Roongbenjawan and Siriphorn, 2020). The number of stands completed was recorded as the score. The highest of the two attempts was recorded.

The handgrip strength was measured using a hand dynamometer. The participant undertook the assessment while seated with their arm resting on the armrest of a chair. The best of three attempts was recorded.

### Physical assessments via telehealth

Due to the limitations of the telehealth format conducted via videoconferencing, anthropometric data was not measured, and the participants only completed the TUG and CST assessments.

The CST was performed as described above. This assessment has been shown to be an acceptable measure when used via telehealth (Peyrusqué et al., 2022).

For the TUG test, the research team mailed the participants a 3-m-long ribbon to measure the walking distance for the test. Participants were advised to show the ribbon taut and laid flat parallel to the walking path. The best of three attempts was recorded. Virtual TUG assessments have been found to be comparable to face-to-face assessments (Karim et al., 2023).

### Questionnaires

The UCLA 3-item Loneliness Scale is a self-reported questionnaire that measures three dimensions of loneliness; relational connectedness, social connectedness, and self-perceived isolation. The three items are measured on a three-point Likert scale with scores ranging from 3 to 9 with higher scores representing greater loneliness (Matthews et al., 2022). The UCLA 3-item Loneliness Scale has been widely used and has been shown to have good validity, with a high convergent validity score (0.82) when compared to the 20 question R-UCLA scale, and to be reliable with an acceptable internal consistency score ( = 0.72; Hughes et al., 2004).

The Social Connectedness Scale (SCS) is a self-reported questionnaire that assesses how connected individuals feel to other people in their social environment. There are eight items that are measured on a six-point Likert scale with scores ranging from 8 to 48. A higher score indicates a greater level of connectedness to others. The SCS has been reported to be reliable with a high score in both internal consistency (0.91) and test-retest reliability (0.96) and valid with a high incremental fit index (0.92) (Lee and Robbins, 1995, 1998).

The Patient Health Questionnaire-9 (PHQ-9) is a self-reported questionnaire that screens for depression. The nine items are measured on a four-point Likert scale and scores can range from 0 to 27. Higher scores denote a greater severity of depression. The PHQ-9 is a validated measure with good sensitivity (0.88) and specificity (0.88) and reported to be reliable with a high internal consistency score (0.86–0.89) and test-retest reliability (0.84) when detecting and monitoring for depression (Kroenke et al., 2010).

### Statistical analysis

Descriptive results were expressed as a median and an interquartile range (IQR) or frequency (percentage). Statistical analysis was performed using Stata 18 (StataCorp LCC, TX, USA). Linear regressions were used to determine associations between waiting times and the SCS, PHQ-9 and the physical assessments results while an ordinal logistic regression was performed for the UCLA questionnaire. The cofounding variables for the analysis were age, sex, education level, OA location (knee, hip, both) and whether OA was unilateral or bilateral. To allow better model fit (determined by visual inspection of the residuals), the waiting time, the PHQ-9 and the TUG were transformed using natural logarithm. Results are expressed as coefficient (or exponentiated coefficient for PHQ-9 and TUG) with 95% confidence intervals, showing the difference in the outcome associated with 10% longer time on the waitlist. Pearson’s correlation coefficient was used to assess the correlation between depression (PHQ-9, log transformed), social connectedness (UCLA score, log transformed) and social connectedness (SCS score). This was performed overall and stratified by sex.

## Results

Eighty participants with lower limb OA participated in the study with 41 tested face-to-face and 39 tested via telehealth. Table 1 displays the demographic characteristics. The mean age of the participants was 63 with an IQR of 58–70. The median wait time was 588 days with an IQR of 205 to 785 days. The mean weight and BMI for the participants was 85.2 kg and 31.4 kg/m^2^ respectively.

**Table 1.** Participant demographic characteristics.

Variable	
Age (years) – median (interquartile range (IQR))	63 (58–70)
Wait time (days) – median (IQR)	588 (205–785)
Joint affected – *n*	
Knee	63
Hip	15
Both knee and hip	2
Bilateral	37
Unilateral	42
Gender – *n* (%)	
Female	42 (52.50%)
Male	37 (46.25%)
Other	1 (1.25%)
Marital status	
Single	12 (15.00%)
Divorced/Separated	15 (18.75%)
Married/Defacto	47 (58.75%)
Widow	6 (7.50%)
Employment – *n* (%)	
Employed	32 (40.00%)
Home duties	10 (12.50%)
Unemployed	38 (47.50%)
Highest education – *n* (%)	
Below year 12	31 (39.00%)
Year 12/Technical and Further Education (TAFE)	17 (22.00%)
Certificate/Diploma	12 (15.00%)
University/Postgraduate	10 (13.00%)
Unknown	9 (11.00%)
Country of birth – *n* (%)	
Australia	44 (55.00%)
Country other than Australia	36 (45.00%)

## Associations between wait time, psychosocial measures and physical assessments

The median scores and the IQRs for each of the psychosocial measures and the physical assessments are presented in Table 2. The unadjusted and adjusted coefficients for the linear regressions performed for the associations between waiting time and the questionnaires and physical assessments are described in Table 3.

**Table 2.** Median total score and interquartile range (IQR) for the psychosocial measures and physical assessments.

Variable	Median total score (IQR)
PHQ-9 (depression scores)	6 (2–10)
Social connectedness scale	30 (25–33)
UCLA loneliness scale	5 (3–6)
Physical assessments	
Timed up and go (s)	8.32 (6.89–10.43)
Chair stand test (n)	11 (8–13)
Handgrip strength (kg)	34 (24.50–42.00)
6-minute walk test (m)	319 (242–394)

**Table 3.** Association between days on waitlist and social connectedness scale, UCLA 3-item loneliness scale, patient health questionnaire-9 and physical function assessments.

Variables	Unadjusted		Adjusted for confounders	
	β [95% CI]	*p* value	β [95% CI]	*p* value
Social connectedness scale (SCS)				
Days on waitlist (increase of 10%)	−0.09 [−0.25, 0.08]	0.299	−0.14[−0.34, 0.05]	0.142
Age			0.25 [0.07, 0.44]	0.008*
Female vs Male			0.66 [−2.34, 3.66]	0.662
Below Year 12			−0.36 [−4.30, 3.59]	0.858
Year 12/TAFE			Ref	
Certificate/Diploma			1.88 [−3.28, 7.04]	0.469
University/Postgraduate			−0.14 [−5.55, 5.27]	0.959
Unknown			2.65 [−2.93, 8.23]	0.347
Hip			Ref	
Knee			0.1 [−4.01, 4.22]	0.96
Hip and Knee			−3.51 [−13.52, 6.50]	0.486
Bilateral			2.27 [−0.72, 5.26]	0.134
Loneliness (UCLA)				
Days on waitlist (increase of 10%)	1.01 [0.97, 1.06]	0.299	1.04 [0.99, 1.10]	0.143
Age			0.93 [0.88, 0.98]	0.008*
Female vs Male			2.79 [1.16, 6.72]	0.022*
Below Year 12			1.25 [0.42, 3.75]	0.685
Year 12/TAFE			Ref	
Certificate/Diploma			0.39 [0.09, 1.79]	0.229
University/Postgraduate			1.57 [0.33, 7.41]	0.568
Unknown			0.09 [0.02, 0.57]	0.01*
Hip			Ref	
Knee			0.45 [0.14, 1.45]	0.182
Hip and Knee			0.57 [0.05, 6.97]	0.658
Bilateral			0.71 [0.30, 1.65]	0.424
Depression (PHQ-9)				
Days on waitlist (increase of 10%)	1.03 [1.01, 1.06]	**0.015** *	1.03 [1.00, 1.06]	0.028*
Age			0.98 [0.95, 1.00]	0.065
Female vs Male			1.8 [1.17, 2.77]	0.008*
Below Year 12			1.03 [0.58, 1.82]	0.915
Year 12/TAFE			Ref	
Certificate/Diploma			0.82 [0.39, 1.72]	0.597
University/Postgraduate			0.93 [0.43, 2.03]	0.86
Unknown			0.53 [0.24, 1.17]	0.114
Hip			Ref	
Knee			0.97 [0.53, 1.75]	0.912
Hip and Knee			1.45 [0.34, 6.15]	0.605
Bilateral			1.13 [0.73, 1.74]	0.573
Physical assessments				
Time up and go (TUG)				
Days on waitlist (increase of 10%)	1.01 [0.99, 1.02]	0.227	1.00 [0.99, 1.01]	0.464
Age			1.01 [1.00, 1.02]	0.106
Female vs Male			1.29 [1.09, 1.52]	0.003*
Below Year 12			1.07 [0.86, 1.34]	0.513
Year 12/TAFE			Ref	
Certificate/Diploma			0.94 [0.71, 1.24]	0.645
University/Postgraduate			0.94 [0.70, 1.26]	0.666
Unknown			0.92 [0.68, 1.24]	0.578
Hip			Ref	
Knee			1.29 [1.03, 1.61]	0.026*
Hip and Knee			0.98 [0.58, 1.67]	0.946
Bilateral			0.95 [0.81, 1.12]	0.544
Chair stand time (CST)				
Days on waitlist (increase of 10%)	−0.03 [−0.14, 0.07]	0.517	0.05 [−0.07, 0.16]	0.424
Age			−0.12 [−0.24,−0.01]	0.033*
Female vs Male			−1.68 [−3.51, 0.14]	0.07
Below Year 12			0.19 [−2.21, 2.60]	0.872
Year 12/TAFE			Ref	
Certificate/Diploma			1.6 [−1.54, 4.74]	0.312
University/Postgraduate			1.42 [−1.87, 4.71]	0.392
Unknown			0.88 [−2.52, 4.28]	0.607
Hip			Ref	
Knee			−1.46 [−3.96, 1.05]	0.25
Hip and Knee			1.21 [−4.88, 7.30]	0.693
Bilateral			−0.51 [−2.33, 1.31]	0.577
Handgrip strength				
Days on waitlist (increase of 10%)	−0.28 [−0.68, 0.11]	0.151	0.04 [−0.33, 0.40]	0.831
Age			−0.18 [−0.60, 0.24]	0.393
Female vs Male			−16.67 [−22.55, −10.79]	<0.001
Below Year 12			−0.9 [−7.42, 5.62]	0.78
Year 12/TAFE			Ref	
Certificate/Diploma			0.92 [−13.17, 15.01]	0.895
University/Postgraduate			−0.85 [−10.44, 8.74]	0.857
Unknown			−2.44 [−20.97, 16.09]	0.79
Hip			Ref	
Knee			−8.87 [−16.95,−0.78]	0.033*
Hip and Knee			−7.49 [−25.90, 10.93]	0.412
Bilateral			5.54 [−0.80, 11.88]	0.084
6-Minute walk test (6MW)				
Days on waitlist (increase of 10%)	−2.84 [−6.47, 0.80]	0.122	−1.73 [−6.57, 3.12]	0.472
Age			−1.73 [−7.37, 3.90]	0.534
Female vs Male			−62.27 [−141.64, 17.09]	0.119
Below Year 12			1.2 [−86.08, 88.48]	0.978
Year 12/TAFE			Ref	
Certificate/Diploma			71.27 [−116.50, 259.04]	0.443
University/Postgraduate			44.85 [−83.00, 172.71]	0.478
Unknown			55.45 [−191.25, 302.16]	0.649
Hip			Ref	
Knee			13.18 [−94.48, 120.85]	0.804
Hip and Knee			29.46 [−215.68, 274.59]	0.807
Bilateral			27.96 [−56.99, 112.91]	0.506

Note. CI = Confidence interval, asterisks indicate statistically significant results.

A 10% longer time on the waitlist was associated with a 3% higher PHQ-9 score both unadjusted (β = 1.03, 95% CI: 1.01, 1.06, *p* = 0.015) and adjusted for age, gender, OA location (knee, hip, both or bilateral) and education (β = 1.03, 95% CI: 1.00, 1.06, *p* = 0.028). The multivariable analysis reported that the female sex was associated with an 80% higher PHQ-9 score (β = 1.8, 95% CI: 1.17, 2.77, *p* = 0.008) while being higher in age was associated with a lower PHQ-9 score (β = 0.98, 95% CI: 0.95, 1.00, *p* = 0.065).

The unadjusted (β = −0.09, 95% CI: −0.25, 0.08, *p* = 0.299) and adjusted (β = −0.14, 95% CI: −0.34, 0.05, *p* = 0.142) linear regressions found no relationship between waiting time and SCS score. The multivariable analysis reported that age was the only confounder associated with higher SCS score (β = 0.25, 95% CI: 0.07, 0.44, *p* = 0.008), indicating that an increase in age of 1 year was associated with a 0.25 point increase in SCS score.

No relationship was found between days on the waitlist and UCLA score when unadjusted (β = 1.01, 95% CI: 0.97, 1.06, *p* = 0.299). The multivariable analysis reported that a lower loneliness score was observed in those at a higher age (β = 1.04, 95% CI: 0.88, 0.98, *p* = 0.008). Further, females are more likely to have higher UCLA score compared to males (β = 2.79, 95% CI: 1.16, 6.72, *p* = 0.022).

### Physical assessments

No association was found between waiting time and TUG (β = 1.01, 95% CI: 0.99, 1.02, *p* = 0.227). The multivariable analysis reported that female identifying people had a 30% longer TUG (β = 1.29, 95% CI: 1.09, 1.52, *p* = 0.003). Additionally, participants with knee OA have 30% longer TUG compared to hip OA (β = 1.29, 95% CI: 1.03, 1.61, *p* = 0.026).

No association was found between waiting time and CST (β = −0.03, 95% CI: −0.14, 0.07, *p* = 0.517). The multivariable analysis reported that female identifying people had 1.7 less stands during the CST test than those identifying as male (β = −1.68, 95% CI: −3.51, 0.14, *p* = 0.07).

No association was found between waiting time and handgrip strength (β = −0.28, 95% CI: −0.68, 0.11, *p* = 0.151). The multivariable analysis reported on average, female identifying people were 17 kg weaker than male identifying people (β = −16.67, 95% CI: −22.55, −10.79, *p* < 0.001). Further, participants with knee OA were 9 kg weaker than participants with hip OA (β = −8.87, 95% CI: −16.95, −0.78, *p* = 0.033), while participants with OA in bilateral joints tended to have slightly stronger grip strength at 5.5kg (β = 5.54, 95% CI: −0.80, 11.88, *p* = 0.084).

No relationship was found between waiting time and the 6MWT (β = −2.84, 95% CI: −6.47, 0.80, *p* = 0.122), nor between any of the confounding variables through the multivariable analysis.

### Relationships between loneliness, social connectedness and depression

A moderate correlation was found between each of the factors; loneliness, social connectedness and depression (Table 4). Depression and loneliness had a positive relationship. Social connectedness had a negative relationship with both depression and loneliness. Loneliness was found to have a stronger correlation with social connectedness and depression in females compared to males, whereas the relationship between depression and social connectedness was similar in both females and males.

**Table 4.** Correlation coefficients for loneliness, social connectedness and depression.

Psychosocial measures	Pearson’s correlation coefficient	*p*-Value
Depression vs loneliness	0.582	<0.001*
Female	0.625	<0.001*
Male	0.499	0.002*
Depression vs social connectedness	−0.470	<0.001*
Female	−0.498	0.001*
Male	−0.525	0.001*
Loneliness vs social connectedness	−0.521	<0.001*
Female	−0.822	<0.001*
Male	−0.249	0.138

Note. Asterisks indicate statistically significant results.

## Discussion

The present study aimed to investigate the relationship between wait times and physical function, loneliness, social connectedness, and depression in individuals with lower limb OA on the waitlist for orthopaedic services at a public hospital. Depression scores was the only outcome found to be associated with wait time, with a 10% longer time on the waitlist being associated with a 3% increase in depression scores. When accounting for confounding factors of age, sex, OA location (knee, hip, both or bilateral) and education level, it was identified that the female sex was associated with greater depression and loneliness, while a higher age was associated with greater social connectedness and lower loneliness.

A novel finding of the current study was that wait time was not associated with reduced physical function. This finding contrasts reports that physical limitations and disability worsen while waiting for treatment (Bachrach-Lindström et al., 2008; Desmeules et al., 2010; Ho et al., 2021; Lizaur Utrilla et al., 2016; McHugh et al., 2008). A possible explanation for the conflicting results is that the present study collected data on physical function through clinical and objective measures conducted by a physiotherapist as opposed to self-reported questionnaires, most commonly being the WOMAC. There can be a discordance between self-reported and objective performance assessments, with studies suggesting that the WOMAC has only a weak to moderate correlation with objective function (Onodera et al., 2020; Selzer et al., 2022). Further studies suggest pain as a influencing factor as individuals with higher pain self-report lower physical function when compared to the objective outcomes (Terwee et al., 2006; Wilfong et al., 2020). While a strength of this study is the inclusion of objective measures, how individuals perceive their physical function and health status can influence how they manage their condition (Jia and Jackson, 2016). Despite not observing a relationship between physical function and waiting time, individuals with lower limb OA in this study lived with physical limitations for a median of 1.6 years while waiting for treatment, which could negatively impact their quality of life (Atukorala and Hunter, 2023).

The findings that longer waitlist duration was associated with greater depression scores aligns with existing literature. A prospective study showed an increase in the rates of depression when on the waitlist for more than 6 months (Lizaur Utrilla et al., 2016). Deterioration in mental health outcomes was observed in further studies with wait times ranging from over 6 months to 2 years (Addai et al., 2024; Ho et al., 2021; Karayiannis et al., 2023). The median wait time for the participants in the current study is approximately 19 months. Across Australia, the median wait time to access a total hip or knee replacement was approximately 14 and 15 months, respectfully (Australian Institute of Health and Welfare, 2023). This indicates that both the sample in this study and individuals with OA on waiting lists nationally are at risk of being affected by depression. The presence of depression and loneliness has been associated with poorer physical and psychological health including greater pain intensity, lower physical function, less social participation, lower self-efficacy, and can be a barrier to engaging with physical activity (Ahn et al., 2024; Alabajos-Cea et al., 2021, 2021; Fonseca-Rodrigues et al., 2022; Pascoe et al., 2024; Smith, 2017; Stubbs et al., 2016; Uritani et al., 2023). Our findings that the female sex and younger age being additional risk factors for depression and loneliness is consistent with the literature (Graham et al., 2024; Wood et al., 2016; Zheng et al., 2021). As lengthy wait times can negatively impact the wellbeing of individuals with OA, interventions that target depression and mental health should be accessible while on the waitlist.

The secondary aim of the study was to investigate the inter-relationship between loneliness, social connectedness and depression and were all found to have moderate correlations in this study. These factors having an interconnected relationship is consistent with the existing literature (Alabajos-Cea et al., 2021; Bevilacqua et al., 2021; Gignac et al., 2013; Luo et al., 2012; Rosemann et al., 2007; Santini et al., 2020; Zheng et al., 2021). Loneliness and depression can be mutually reinforcing, as withdrawing from socialisation can lead to depressive symptoms and vice versa, while social connectedness can act as a buffer against loneliness and depression. Therefore addressing the modifiable risk factors of OA concurrently may result in the greatest improvements (Ahn et al., 2024; Burley et al., 2023). A meta-analysis exploring non-pharmacological approaches to OA management identified that movement meditation, including yoga, tai chi, or qigong, and multimodal approaches, including a culmination of exercise, psychological therapy, and education in one intervention, were found to be the most beneficial in reducing depressive symptoms (Burley et al., 2023). Additionally, a systematic review uncovered that physical activity interventions can lead to a reduction in loneliness, with in-person group exercise programs showing the most promise (Ahn et al., 2024). Interestingly, a study attempting to improve social support also found an increase in self-reported exercise post-intervention further indicating that addressing one aspect of OA can lead to improvements in other areas (Geffen et al., 2019).

A potential limitation of the current study is the relatively small sample size. Additionally, restrictions due to the COVID-19 pandemic required the physical assessments to be conducted online for half of the participants and further limited the ability to collect data. Further, the present study focused on outcomes that could have been influenced by the COVID-19 pandemic. Moreover, confounding variables, including length of symptom duration and pain severity were not collected, which can serve as a limitation. The cross-sectional study design may also be a limitation, particularly, given the potential impacts of wait time on health status.

## Conclusion

In conclusion, a greater length of time on the waitlist was associated with a higher depression score. However, time on the waitlist was not associated with lower physical function and social connectedness, nor with greater loneliness. Mental health interventions that target depression should be provided to patients on the waitlist for orthopaedic services, particularly, interventions that also encourage social connectedness to buffer potential loneliness. Future research would benefit from longitudinal studies that collect data beyond the 12-month time point as the average Australia patient is on the waitlist for orthopaedic services for more than a year.
